# Improving quality of care for maternal and newborn health: a pre-post evaluation of the Safe Childbirth Checklist at a hospital in Bangladesh

**DOI:** 10.1186/s12884-017-1588-x

**Published:** 2017-12-04

**Authors:** Herfina Y. Nababan, Rubana Islam, Shabnam Mostari, Md Tariqujjaman, Malabika Sarker, Mohammad Tajul Islam, Corrina Moucheraud

**Affiliations:** 1 Centre for Universal Health Coverage, Health Systems and Population Studies Division, International Centre for Diarrhoeal Disease Research, 68 Shahid Tajuddin Ahmed Sharani, Mohakhali, Dhaka, 1212 Bangladesh; 2 Nossal Institute for Global Health, Melbourne School of Population and Global Health, the University of Melbourne, 333 Exhibition Street, Melbourne, Victoria 3004 Australia; 3 School of Public Health & Community Medicine, Faculty of Medicine, University of New South Wales, Sydney, Australia; 4 James P. Grant School of Public Health, BRAC University, 68 Shahid Tajuddin Ahmed Sharani, Mohakhali, Dhaka, 1212 Bangladesh; 5 Japan International Cooperation Agency (JICA), 3rd Floor, Bay’s Galleria, 57 Gulshan Avenue (CWS-A19), Gulshan-1, Dhaka, 1212 Bangladesh; 6 Department of Health Policy and Management, UCLA Fielding School of Public Health, 650 Charles E Young Dr S, Los Angeles, CA 90095 USA

**Keywords:** Maternal and newborn health, Safe Childbirth Checklist, Quality of care, Health worker performance, Health service delivery, Bangladesh

## Abstract

**Background:**

Bangladesh has achieved major gains in maternal and newborn survival, facility childbirth and skilled birth attendance between 1991 and 2010, but excess maternal mortality persists. High-quality maternal health care is necessary to address this burden. Implementation of WHO Safe Childbirth Checklist (SCC), whose items address the major causes of maternal deaths, is hypothesized to improve adherence of providers to essential childbirth practices.

**Method:**

The SCC was adapted for the local context through expert consultation meetings, creating a total of 27 checklist items. This study was a pre-post evaluation of SCC implementation. Data were collected over 8 months at Magura District Hospital. We analysed 468 direct observations of birth (main analysis using 310 complete observations and sensitivity analysis with the additional 158 incomplete observations) from admission to discharge. The primary outcome of interest was the number of essential childbirth practices performed before compared to after SCC implementation. The change was assessed using adjusted Poisson regression models accounting for clustering by nurse-midwives.

**Result:**

After checklist introduction, significant improvements were observed: on average, around 70% more of these safe childbirth practices were performed in the follow-up period compared to baseline (from 11 to 19 out of 27 practices). Substantial increases were seen in communication between nurse-midwives and mothers (counselling), and in management of complications (including rational use of medicines). In multivariable models that included characteristics of the mothers and of the nurse-midwives, the rate of delivering the essential childbirth practices was 1.71 times greater in the follow-up compared to baseline (95% CI 1.61–1.81).

**Conclusion:**

Implementation of SCC has the potential to improve essential childbirth practice in resource-poor settings like Bangladesh. This study emphasizes the need for health system strengthening in order to achieve the full advantages of SCC implementation.

**Electronic supplementary material:**

The online version of this article (10.1186/s12884-017-1588-x) contains supplementary material, which is available to authorized users.

## Background

The end of the Millennium Development Goals (MDGs) era offers an opportunity to examine successes and failures in global health and development, and to identify priorities for the future [1]. Overall, the world achieved major gains in maternal and child survival and in coverage of facility-based childbirth and skilled birth attendance [2, 3]. Many women and newborns, however, continue to die during and shortly after childbirth, and disparities persist across countries as well as across groups within countries [1, 2]. This suggests that the global strategy, which has focused on increasing coverage of skilled birth attendance, did not eliminate all excess burden [4]. Utilization alone is not sufficient; high-quality maternal health care is essential for reducing excess mortality [5, 6].

Mirroring global trends, Bangladesh made great progress towards MDG 4 and 5 targets [1]. While the current neonatal mortality rate of 28 per 1000 live births has been lower than the target of 48 deaths per 1000 live births, its maternal mortality ratio (MMR) decreased from 574 maternal deaths per 100,000 live births in 1991 to 176 deaths per 100,000 live births in 2015 [7, 8]; and use of facility delivery increased over this period [9]. However, there is an unfinished agenda for improving childbirth outcomes in Bangladesh. Still only 37% of births occur at health facilities, with much lower rates among poorer women, and provision of quality care during childbirth remains a particular challenge [9–11].

High-quality childbirth care requires facility readiness (physical infrastructure, equipment, medicines and supplies) and availability of human resources with the knowledge, skills, and capacity to effectively manage uncomplicated and complicated childbirth events [12]. In 2008, World Health Organization (WHO) designed the Safe Childbirth Checklist (SCC), a 29-item quality improvement tool to help reduce adverse childbirth events by facilitating frontline health workers’ adherence to evidence-based essential childbirth practices [13]. The items on the SCC address the major causes of maternal deaths, intrapartum-related stillbirths and early neonatal deaths [13].

In the current study, we examined the effectiveness of the SCC in improving childbirth practices at a district hospital in Bangladesh. The use of SCC is hypothesized to facilitate adherence to essential childbirth practices demonstrated to be effective in reducing maternal and newborn deaths, by reminding nurse-midwives to perform the practices and in the proper sequence [13]. The results from this study were expected to expand the emerging body of empirical evidence about the SCC – including evaluation results from India and Sri Lanka [14, 15].

## Methods

### Study setting

This study was conducted at a 100-bedded district level secondary care hospital (Magura District Hospital (DH)) in Khulna division in Southwestern Bangladesh, around 260 km from the capital Dhaka. This hospital was purposively selected because it sees a high annual volume of births (on average more than 1200 uncomplicated vaginal deliveries per year) and was implementing no other safe childbirth interventions during this evaluation period [16]. It also had all requisite supplies and materials to perform the SCC activities, except the partograph and disposable surgical gloves which were provided for the purposes of this study for the entire study period.

### Adaptation of the SCC

There was a series of consultative meetings between study investigators and external experts (obstetricians and neonatologists from Bangabandhu Sheikh Mujib Medical University, Shaheed Suhrawardy Medical College Hospital; and public health scientists and researchers affiliated with BRAC, the International Center for Diarrhoeal Disease Research, Bangladesh (icddr,b), Japan International Cooperation Agency (JICA), and James P. Grant School of Public Health – BRAC University) to reach consensus on how to adapt the SCC for the local context. This is a recommended first step by WHO for each instance of local implementation [17]. These proposed adaptations were shared with WHO for their review and feedback, and a revised checklist version was pre-tested in February 2014 at the Narsingdi district hospital (Dhaka division), which has characteristics similar to the study hospital. The key differences between the final checklist used in this study and WHO SCC are: removal of HIV-related items (due to low prevalence in Bangladesh); and addition of a question to identify women who had already received oxytocin outside of the hospital. The later was added to prevent double dose in women who were sent to Magura DH by traditional birth attendants or village doctors, given the wide practice of oxytocin at the community level by these providers [18]. In total, there are 27 checklist items adopted in the current study, consisting of practices such as partograph use, checking the need for antibiotics or magnesium sulfate and ensuring the availability of bedside supplies. These practices were organized into four pause points: on admission, just before pushing, soon after birth, and before discharge (See Additional file 1: Table S1 for the full checklist items).

### Study design and data collection

A pre-post study design was used to measure the association between SCC implementation and changes in safe childbirth care. Baseline (pre) data were collected at the obstetric unit of Magura DH between April and June 2014. Then the SCC was introduced: eight nurse-midwives (all with more than 10 years of working experience) responsible for care in delivery ward were trained to use SCC, with special focus on the partograph because this was not routine in Magura DH. They were subsequently requested to use the SCC during all childbirth practices. There were additional orientation sessions on the SCC for obstetricians, health care managers and medical officers working in the obstetric unit. The follow-up (post) phase began in July 2014, with 5 months of data collection (ending in November 2014).

The sample size for the active intervention phase was calculated at a power of 80% and significance level of 5%. The formula was used assuming a 150% increase in partograph use, given the extremely low rate of current practice (5%) [19]. The total sample size calculated was 440 i.e. 220 deliveries for each phase of the study. Only uncomplicated vaginal deliveries admitted before the active stage of labor was observed. Pregnant women who required Caesarean birth were excluded from the study, as well as pregnant women who were HIV positive.

Data at baseline and follow-up were collected by three research assistants who were nursing school graduates with working experience in obstetric care, with supervision on-site provided by a retired senior nurse. The data collectors received extensive preparatory training on the study objectives, data collection procedure and skill, informed consent, as well as on the SCC and evidence-based essential practices incorporated in the SCC. To ensure that observation was done in a similar manner, a pilot data collection was conducted in a different district hospital. Data were collected via direct observation of birth events to record providers’ behavior. All births were observed for 24 h per day for 6 days each week except during official holidays, for all the eight nurse-midwives at Magura DH. Research assistants worked an eight-hour shift (including the night shift) and then handing the observation over to the next research assistant in duty when required.

### Data analysis

The primary outcome of interest was the number of essential childbirth practices—operationalized as items on the SCC—performed per childbirth event. We tested how this was associated with the SCC intervention by performing statistical tests of difference (Wald test) between births occurring during baseline and follow-up periods. Additionally, we examined whether other factors were related to childbirth practices, comprising of mothers’ characteristics (age, parity, and number of antenatal care (ANC) visits completed which were treated as continuous variables, and educational attainment which was categorized into no education/primary, secondary and higher) and nurse-midwives’ characteristics (age, years of experience in healthcare, and months after the last refresher training; all treated as continuous variables). This was tested using multivariable Poisson regression models with reported Incidence Rate Ratios (IRR), accounting for clustering by nurse-midwives. The first multivariate model was unadjusted, the second model included covariates for mothers, and the third model included covariates for mothers and nurse-midwives. The main analysis was done using complete observations i.e. childbirth events whose all 100% of pause points were observed. However, the incomplete observations were incorporated for the sensitivity analysis. Data were entered and verified with SPSS version 20. Analyses were performed with Stata version 13 (Stata Corp.).

## Results

The analysis included 310 complete observations of childbirth events from Magura DH, comprising of 153 and 157 observations at baseline and endline respectively. Characteristics of the mothers represented in this sample are presented in Table 1. The majority of the women were between 20 and 29 years old and had given birth to their first child. There were no statistically significant differences between characteristics of women examined at baseline and endline.

Additional file 2 Table S2 includes information about the eight nurse-midwives from Magura DH who provided care for the births in this sample. All nurse-midwives had at least 10 years of experience, and three had recently received refresher training on childbirth care. The numbers of childbirth observation of each nurse-midwife varied (See Additional file 2: Table S2), likewise the numbers of observation completed by each observer also varied (See Additional file 3: Table S3).

**Table 1:** Demographic characteristics of mothers at baseline and endline

Characteristics	Baseline (*N* = 153)	Endline (*N* = 157)	*P*-Value
	n (%)	n (%)	
Age (years)			
< 20	19 (12)	17(11)	0.156
20–24	66 (43)	73(47)	
25–29	48 (31)	35(22)	
≥ 30	20 (13)	32 (20)	
Median age (IQR) (years)	23 (20, 25)	23 (20, 28)	0.52
Education			
Primary or no education	45 (29)	57 (36)	0.122
Secondary	85 (56)	69 (44)	
Higher	23 (15)	31 (20)	
Parity			
0	84 (55)	84 (54)	0.941
1	44 (29)	48 (31)	
≥ 2	25 (16)	25 (16)	
Number of ANC visit			
0	25 (16)	17 (11)	0.27
1	82 (54)	96 (61)	
≥ 2	46 (30)	44 (28)	

Following implementation of the SCC, the average number of safe childbirth practices performed per childbirth increased by approximately 70%, from 11 out of 27 practices in baseline to 19 out of 27 practices in endline (See Table 2). This was a statistically significant increase according to a series of multivariable Poisson regression models that included covariates for mothers’ and nurse-midwives’ characteristics (See Table 3). The result was robust for all specifications, with the rate of delivering the essential childbirth practices being 1.71 times greater in the follow-up compared to baseline (95% CI 1·61–1·82), and no covariates were found significant.

**Table 2:** Number of essential childbirth practices (SCC items) performed at baseline and endline (complete observations)

Childbirth practices	Baseline (*N* = 153)	End line (*N* = 157)	*P*-value
	n (%)	n (%)	
On admission			
Partograph use	0 (0)	0 (0)	–
Appropriate maternal infection management	1 (0.65)	149 (95)	<0.001
Appropriate eclampsia management	6 (4)	129 (82)	<0.001
Hand hygiene	152 (99)	157 (100)	0.318
Birth companion present	148 (97)	157 (100)	0.024
Intrapartum counseling	3 (2)	121 (77)	<0.001
Just before pushing			
Emergency birth helper was identified	152 (99)	156 (99)	0.99
Appropriate maternal infection management	1 (0.65)	147 (94)	<0.001
Appropriate eclampsia management	4 (3)	125 (80)	<0.001
Supplies for mothers and babies	0 (0)	18 (11)	<0.001
Soon after birth (within 1 h)			
AMTSL	134 (88)	156 (99)	<0.001
Appropriate newborn thermal and resuscitation	17 (11)	0 (0)	<0.001
Maternal blood loss assessment and treatment	152 (99)	157 (100)	0.318
Appropriate maternal infection management	3 (2)	148 (94)	<0.001
Appropriate eclampsia management	3 (2)	119 (76)	<0.001
Newborn referral	149 (97)	157 (100)	0.044
Appropriate newborn infection management	0 (0)	0 (0)	–
Newborn special care	0 (0)	0 (0)	–
Breastfeeding and skin to skin contact	152 (99)	157 (100)	0.318
Postpartum counseling	3 (2)	133 (85)	<0.001
Before discharge			
Maternal blood loss assessment	152 (99)	157 (100)	0.318
Appropriate maternal infection management	0 (0)	35 (22)	<0.001
Appropriate newborn infection management	1 (0.65)	1 (0.64)	0.985
Newborn feeding assessment	152 (99)	157 (100)	0.318
Family planning counseling	152 (99)	157 (100)	0.318
Discharge counseling	1 (0.65)	108 (69)	<0.001
Follow-up visit counseling	151 (99)	157 (100)	0.156
Median total practices performed	11 (41)	19 (70)	<0.000

The *P*-values were generated from Wald tests

**Table 3:** Multivariable Poisson regression models assessing factors associated with improvement in essential childbirth practices

Variable	Model (1)	Model (2)	Model (3)
	IRR (95%CI)	IRR (95%CI)	IRR (95%CI)
WHO SCC use			
Yes (endline)	1.71^a^	1.70^a^	1.71^a^
	(1.68-1.73)	(1.68–1.73)	(1.61–1.82)
Nurse-midwives’ age^b^			1
			(1.00-1.03)
Nurse-midwives’ year of experience^b^			0.96
			(0.94–0.98)
Months since nurse-midwives’ last refresher training^b^			1
			(0.98-1.02)
Mothers’ age^b^		1	1
		(1.00-1.03)	(1.00–1.03)
Mothers’ education background			
Primary or no education		(ref)	(ref)
Secondary		0.99	0.99
		(0.97–1.01)	(0.97–1.01)
Higher		1	1
		(0.96–1.03)	(0.96–1.03)
Parity^b^		1	1
		(0.98-1.02)	(0.98–1.02)
Number of ANC visit^b^		1	1
		(0.99-1.02)	(0.99–1.02)
Constant	11.04	11.2	11.33
	(10.92–11.16)	(10.18–12.32)	(9.98–12.86)
Number of observations	310	310	310

Outcome variable is number of SCC practices performed per childbirth

Model (1) includes no covariates; Model (2) includes covariates for the mothers; Model (3) includes these plus covariates for the nurse-midwives

^a^denotes significance at the 1% level

^b^indicates a continuous variable

Some checklist items related to appropriate management of maternal infection and of eclampsia as well as appropriate newborn infection management were composite measures representing a series of sub-actions, consisting of checking for symptoms and giving the medicine when needed (See Additional file 1: Table S1 for the full checklist items). In the absence of any symptom, it was expected that the nurse-midwives would not give the medicine. Following SCC implementation, the largest improvement for these items for mothers was in the first component i.e. checking for symptoms (Table 4). Provision of antibiotics and of magnesium sulfate at endline was more likely to occur after symptoms were checked, although at endline 89% of the mothers who were prescribed antibiotics at discharge had been checked for symptoms and no symptoms were present.

**Table 4:** Appropriate use of medicines at baseline and endline

	Received medicine, n		Had any symptoms checked, n		Had any symptoms present, n	
	Baseline (*n* = 153)	Endline (*n* = 157)	(as % of those who received medicine)		(as % of those who had symptoms checked)	
Maternal antibiotics						
On admission	0	1	0	1 (100%)	0	1 (100%)
Just before/after birth	0	1	0	1 (100%)	0	1 (100%)
One hour postpartum	4	3	0 (0%)	3 (100%)	0	1 (33%)
Before discharge	90	111	2 (2.2%)	99 (89%)	0 (0%)	0 (0%)
Magnesium sulfate						
On admission	2	2	0 (0%)	2 (100%)	0	1 (50%)
Just before/after birth	2	2	0 (0%)	1 (50%)	0	1 (100%)
One hour postpartum	2	2	0 (0%)	2 (100%)	0	2 (100%)

When examining the improvements seen on specific activities (See Table 2), maternal counseling on admission, soon after birth, and before discharge improved considerably, from near-zero rates to near-universal coverage by endline. Statistically significant improvements were also seen in the active management of the third stage of labor (AMTSL) (from 88% to 99%) and the bedside availability of supplies for mothers and babies such as oxytocin 10 IU in syringe and sterile blade to cut the cord (from 0% to 11%). Only two women received oxytocin at the community level prior to admission (results not shown).

Some safe childbirth practices were already commonly performed at baseline, such as hand hygiene (99%), encouraging the presence of a birth companion (97%), assessing and treating postpartum maternal blood loss (99%), breastfeeding and skin-to-skin contact soon after birth (99%), newborn feeding assessment (99%), and counseling for family planning and for a follow-up visit (99% each). Other behaviors were not performed at baseline and did not improve with introduction of the SCC: partograph use (0% at baseline and endline), appropriate management of newborn infection (1% at baseline and endline), and checking the need for newborn special care (0% at baseline and endline). Appropriate newborn thermal and resuscitation practices (special care) declined between baseline and endline (from 11% to 0%).

As a sensitivity analysis, we reran the analysis on a dataset that also included incomplete observations (In total 468 observations, with additional 76 and 82 incomplete observations at baseline and endline respectively) (See Additional file 4: Table S4), and all results remained the same.

## Discussion

### Main findings and interpretation

We observed a significant and substantial increase in the number of evidence-based safe childbirth practices following the introduction of the SCC. The magnitude of this improvement is similar to results from a previous evaluation of the SCC in India [14], and the endline results from Sri Lanka [15]. This result is also consistent with previous studies showing that health worker reminders and prompts were associated with improvements in quality care for maternal and child health care in low and middle income countries (LMICs) [20].

Maternal counselling on admission, soon after birth, and before discharge saw considerable improvement in this study. Such counselling is important to ensure that women are treated with respect and are empowered to participate in decisions about their own care [21], which may encourage health service utilization [22, 23]. It has previously been reported that poor provider-patient communication can discourage health service use [24] – so the SCC offers a model for how to improve such exchanges. The improvement in bedside availability of birth supplies is likewise noteworthy, as it is important that medicine and supplies are readily available to providers to encourage the performance of childbirth practices [14]. An important practice that relies upon the availability of supplies was among others the AMTSL.

Some other childbirth practices were already commonly performed at baseline in this study – including breastfeeding and handwashing. A 2014 health facility survey in Bangladesh found that 79% of district hospitals had the requisite supplies for hand hygiene (i.e. soap and running water) [25], which suggests that, where infrastructure and supplies were available, they were being used for safe childbirth care. In line with the current findings, the results from previous study assessing essential newborn practices in Bangladesh also demonstrated that the coverage of practices such as handwashing and breastfeeding were high [26].

Some practices including partograph, appropriate management of newborn infection and checking the need for newborn special care were not performed in the baseline and did not see any improvement after SCC introduction. At the same time, newborn thermal and resuscitation practices declined in the endline. Other than inappropriate attitude, inadequate knowledge and skills can deter good health care [27]. Nonetheless, today it is widely recognized that health workers’ performance is not only a function of these individual characteristics [28]. An enabling environment, managerial culture and organizational context as well as the overall health system also play interrelated crucial roles [28, 29]. Factors including absence of teamwork, high workload, shortage of drugs, unavailability of equipment and supplies, and the lack of supervision and training opportunities can demotivate health workers [27]. Furthermore, factors such as resources allocation, planning and deployment of health workers, policy and regulatory framework, communication and decision-making processes, and accountability mechanisms also influence performance from the broader health system perspective [28].

The non-use of partograph found in this study is disheartening, given its importance in preventing prolonged labor and subsequent possible events such as postpartum haemorrhage, maternal or newborn infection, as well as birth asphyxia [30]. Partograph is a low technology tool that graphically represents key events during labor and is recommended by WHO for routine monitoring of labor to provide an early warning system and take action according to a clinical management protocol [29]. Its underuse is unfortunately often found in LMICs, including Bangladesh [19, 29, 31]. In accordance with health workers’ performance in general, the barrier of its use also ranges from individual, social and organizational contexts [29]. It is perceived as time-consuming [32], especially in the settings with high workload and/or understaffed [33]. Some organizational barriers include shortage of clinical equipment, medicine and supply [32, 34], including unavailability of partograph itself. These underlying issues are often problematic in LMICs, including Magura DH [30]. Training was deemed important to improve the ability of midwives to use partographs [35]. Nevertheless, with training and constant supply, partograph use didn’t improve in the current study. Since partograph was not available and its correct use was not a norm prior to the study, it was probable that no proper enforcement i.e. supervision and monitoring was in place to maintain its use [36]. Consequently, although it was made available for this study and training was provided to the nurse-midwives, a lack of experience and/or low confidence may have limited its uptake [29]. Recognizing the fundamental role of policy and leadership, it is suggested that an implementation strategy should also include policy level interventions to ensure quality assurance and improvement activities including rigorous implementation of management protocols [30, 37].

Other practices that did not improve in the current study were related to newborn care. Twenty-four hours period immediately after birth holds the highest risk of neonatal deaths resulting from conditions such as birth asphyxia, pre-term birth related problems, and neonatal infection, suggesting the need for high quality newborn care [38]. Acknowledging this risk, WHO developed a course to strengthen the capacity of health workers to deliver a set of minimum evidence-based newborn care called the Essential Newborn Care (ENC) that has been proven to improve care for newborns and lead to a decrease in neonatal death [39]. The practices within ENC that address the major causes of neonatal death were also incorporated into SCC [13]. The finding from this study therefore suggest the need to strengthen care in this area if a decline in neonatal death is to be achieved. Improving the competence of nurse-midwives is important given the low numbers of staff in district hospitals in Bangladesh who had ever received training in newborn care and resuscitation. However, improvement in organizational and structural context including adequacy of health workforce, supportive supervision and availability of necessary supply is also necessary [25, 26]. In addition. The decline in newborn thermal and resuscitation practice found in this study following SCC implementation was likely prompted by SCC, showing that these practices should be performed only when the need for these practices were confirmed.

An encouraging finding from this study was the increase in checking for symptoms to ensure appropriate use of medicines to manage maternal infection and eclampsia after implementation of the SCC – especially at discharge, although 89% of women at endline were still given antibiotics despite having no infection symptoms. WHO has found widespread substandard medicine use worldwide [40], which may be due to misinformation, provider and/or patient bias and preference, prescribing habits, peer pressure, system constraints including lack of diagnostics, or economic incentives from the pharmaceutical industry that may promote overuse [41–43]. This study did not explore these hypothesized mechanisms, but other studies from Bangladesh have identified these as salient factors for misuse of medicines locally [44]. It can be difficult to identify women at risk of developing infections during childbirth – which may be more prevalent among women who deliver at health facilities due to unhygienic conditions, poor water and sanitation systems, overcrowding and poor quality care [45] – since symptoms (such as fever and abnormal vaginal discharge) can be non-specific and may manifest only after childbirth [46]. Therefore providers may be overly cautious, and as a result prescribe broad-spectrum antibiotics during childbirth [42]. This may be one reason for the persisting use of antibiotics at discharge in this study; these reasons were echoed in informal discussions with providers at Magura DH about this finding. Irrational medicine use has serious public health and clinical consequences, including increased adverse drug events and accelerating rates of antimicrobial resistance [40]. This study suggests that there is a need for tools to address the issue of dispensing even in the absence of symptoms, for example via continuous quality improvement to encourage rational use [42].

Finally, the result of the Poisson model that included nurse-midwives’ characteristics showed that the number of years of experience was associated with worse childbirth practices, even though this result was not significant. Previous studies have found both positive and negative correlations between experience and health workers’ performance [47–49]. The association could likely be explained by better self-confidence of more experienced nurse-midwives of their clinical experience and intuition which made them less willing to follow a new tool or guideline or less open to new ideas [47]. In line with this, different studies revealed that higher-level cadres of health workers who are usually also more experienced tend to perform less well at following new guidelines compared to their lower level counterparts [50, 51].

### Strength and limitation

A key advantage of this study over previous evaluations was its continuous measurement of SCC activities for each observed childbirth – from admission to discharge. But several limitations should also be noted. First, a randomized trial design – including a control arm (with no SCC implementation) – would have allowed for stronger causal inference in this evaluation, but such an evaluation was beyond the resources available for this study. The current study was also not designed to detect any change in outcome of maternal and neonatal morbidity and mortality. Second, some supplies (i.e. gloves and partograph) were provided by the investigators, to enable the assessment of SCC’ use without supply constraints. These results may therefore not be generalizable to other settings where such supplies are unavailable. Third, the 310 complete observations analyzed in this study were falling short of the sample size calculated of 440 observations. Nonetheless, the sensitivity analysis incorporating incomplete observations didn’t alter the results. Fourth, direct observation of nurse-midwives’ practices could introduce bias, such as a Hawthorne effect whereby performance is improved due to the act of observation. However, the same groups of nurse-midwives were observed pre- and post- intervention, therefore cancelling this effect. Fifth, the findings from the current study may not be generalizable to providers with less than 10 years of experience, providers other than nurse-midwives, or providers working in different settings. Lastly, it was difficult to measure certain SCC items, such as blood loss and management of newborn infection, which involved subjective or otherwise challenging observation. This may have caused some under-reporting, so more detailed measurement guidelines and instructions (as part of WHO SCC program) might be useful for future studies.

## Conclusion

The overall improvement in nurse-midwives’ practices was significant and strong. It suggests that implementing the SCC may facilitate health worker’ compliance to essential childbirth practices. The study has identified areas of practice that should be strengthened in Bangladesh, such as prevention of prolonged labor using partograph as well as essential newborn care. It also pointed out the importance of available workforce, infrastructure and supplies, of strengthening the role of leadership and continuous quality assurance and improvement, as well as of strengthening the overall health system to achieve the full advantages of SCC implementation. The results of SCC implementation evaluation in Bangladesh presented in this paper offers new evidence and insights for expanding use of the SCC in low-income settings.

## Additional files

Additional file 1: Table S1. Final SCC used in the current study. (XLSX 17 kb)

Additional file 2: Table S2. Characteristics of nurse-midwives. (XLSX 13 kb)

Additional file 3: Table S3. Distribution of observation completion. (XLSX 12 kb)

Additional file 4: Table S4. Number of essential childbirth practices (SCC items) performed at baseline and endline (including incomplete observations). (XLSX 14 kb)

## Abbreviation

AMTSL: Active management of the third stage of labor
ANC: Antenatal care
DGHS: Directorate General of Health Services
DH: District hospital
icddr,b: International Center for Diarrhoeal Disease Research, Bangladesh
JICA: Japan International Cooperation Agency
MDGs: Millennium development goals
MMR: Maternal mortality ratio
SCC: Safe Childbirth Checklist
WHO: World Health Organization
